# In Vitro Effects of Dehydrotrametenolic Acid on Skin Barrier Function

**DOI:** 10.3390/molecules24244583

**Published:** 2019-12-14

**Authors:** Eunju Choi, Young-Gyu Kang, So-Hyeon Hwang, Jin Kyeong Kim, Yong Deog Hong, Won-Seok Park, Donghyun Kim, Eunji Kim, Jae Youl Cho

**Affiliations:** 1 Department of Integrative Biotechnology, Sungkyunkwan University, Suwon 16419, Korea; cej223@naver.com (E.C.); sohyun031195@naver.com (S.-H.H.); rosekim95@naver.com (J.K.K.); 2 Basic Research & Innovation Division, R&D Center, AmorePacific Corporation, Yongin 17074, Korea; kangyg82@amorepacific.com (Y.-G.K.); hydhong@amorepacific.com (Y.D.H.); wspark@amorepacific.com (W.-S.P.); dhkim417@amorepacific.com (D.K.)

**Keywords:** skin barrier, skin hydration, keratinocyte differentiation

## Abstract

Dehydrotrametenolic acid (DTA) is a lanostane-type triterpene acid isolated from *Poria cocos* Wolf (Polyporaceae). Several studies have reported the anti-inflammatory and antidiabetic effects of DTA; however, its effects on the skin are poorly understood. In this study, we investigated the effects of DTA on skin barrier function in vitro and its regulatory mechanism in human keratinocyte cell line HaCaT cells. DTA increased the microRNA (mRNA) expression of natural moisturizing factor-related genes, such as HAS-2, HAS-3, and AQP3 in HaCaT cells. DTA also upregulated the mRNA expression of various keratinocyte differentiation markers, including TGM-1, involucrin, and caspase-14. Moreover, the protein expression of HAS-2, HAS-3, and TGM-2 were significantly increased by DTA. To examine the regulatory mechanisms of DTA, Western blotting, luciferase-reporter assays, and RT-PCR were conducted. The phosphorylation of mitogen-activated protein kinases (MAPKs) and IκBα were increased in DTA-treated HaCaT cells. In addition, AP-1 and NF-κB transcriptional factors were dose-dependently activated by DTA. Taken together, our in vitro mechanism studies indicate that the regulatory effects of DTA on skin hydration and keratinocyte differentiation are mediated by the MAPK/AP-1 and IκBα/NF-κB pathways. In addition, DTA could be a promising ingredient in cosmetics for moisturizing and increased skin barrier function.

## 1. Introduction

Skin is an important physical barrier that protects the body from external threats, such as infections, ultraviolet (UV) radiation, and harmful chemicals. It also maintains homeostasis, including body temperature and moisture [1]. The skin consists of the epidermis, dermis, and hypodermis layers. The epidermis is the outermost layer of skin and composed of keratinocytes, Langerhans cells, and melanocytes. The keratinocytes constitute the majority of the cells of the epidermis and produce keratin to form the skin barrier through keratinization [2,3].

Dysfunction of the skin barrier can lead to skin aging and delayed wound healing, as well as severe diseases, such as psoriasis and atopic dermatitis. The most common cause of skin barrier dysfunction is a loss of skin moisture balance regulated by hyaluronic acid (HA) and natural moisturizing factors (NMFs) [4,5]. HA promotes skin hydration and plastic properties of the skin, and is synthesized by hyaluronic acid syntheses (HASs) [6]. Moreover, HA stimulates the cell proliferation and differentiation of keratinocytes. HASs have three isoforms, HAS-1, HAS-2, and HAS-3, and are regulated by cytokines and growth factors, such as the epidermis growth factor (EGF) [7,8]. HAS-2, stimulated by growth factors, induces HA synthesis in skin keratinocytes [9,10]. Also, HAS-3 plays an essential role in the synthesis of hyaluronan in the epidermis [11]. Another important gene in skin hydration is aquaporin 3 (AQP3), one of 13 isotypes of aquaporin transmembrane channels in humans. These molecular water channels are involved in various physiological functions, such as cellular metabolism, water balance, and wound healing through transporting glycerol and water [12,13]. In particular, AQP3 found in the epidermal basal cell layer plays an important role in skin barrier function and facilitates differentiation in keratinocytes [14].

The epidermis maintains balance through the self-renewal of differentiated keratinocytes from the basal layer. The regeneration and repair of the skin are important for it to function properly [15]; thus, keratinocyte differentiation is essential for maintaining the skin barrier. Epidermis barrier formation is associated with various genes, such as involucrin, transglutaminase (TGM), and filaggrin (FLG) [16,17]. In particular, involucrin, a cell envelope protein, is expressed at an early stage of keratinocyte differentiation, and promotes envelope formation and cellular cohesion [18]. Keratinocyte differentiation is importantly modulated by mitogen-activated protein kinase (MAPK) signaling pathways—ERK, JNK, and p38. Specifically, p38 activates involucrin expression, and ERK1/2 kinases enhance proliferation and differentiation of keratinocytes [19,20].

**Figure 1:**
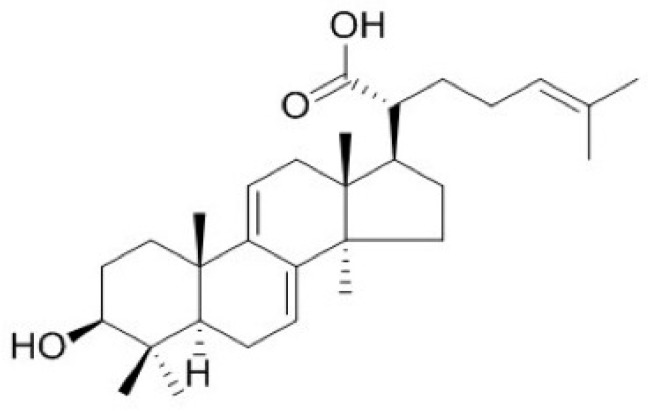
Structure of dehydrotrametenolic acid (DTA).

*Poria cocos* Wolf (Polyporaceae), a rotten pine-tree fungus, is naturally distributed in East Asia, including Korea, China, and Japan. It has traditionally been used as a [21]. Dried sclerotia of *P. cocos* Wolf are widely used to treat various diseases, such as hypertension and diabetes alone, or in combination with other herbal medicines [22,23,24]. Dehydrotrametenolic acid (DTA, Figure 1) is a lanostane-type triterpene acid isolated from the sclerotium of *P. cocos*. Similar lanostane-type triterpene acids have various biological activities, such as anti-inflammation and antidiabetic effects [25,26]. However, the effects of DTA on barrier function of the skin have not been reported. In this study, we investigated the effects of DTA on skin barrier function and its regulatory mechanisms using human keratinocyte cell line HaCaT cells. 

## 2. Results

### 2.1. Effects of DTA on Skin Hydration

**Figure 2:**
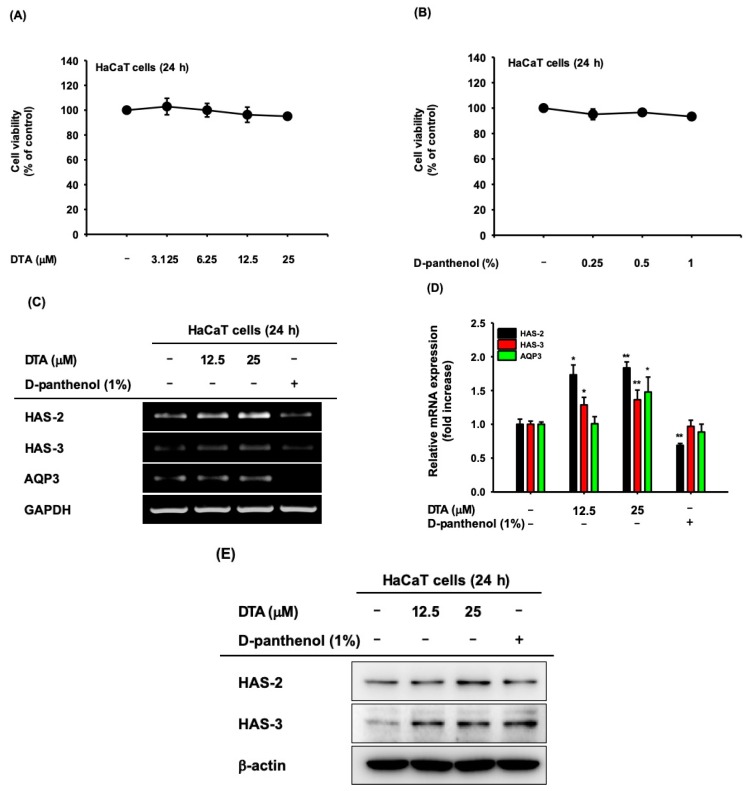
Effects of DTA on skin hydration in human keratinocyte cell line HaCaT cells. HaCaT cells were treated with the indicated concentration of DTA (**A**) or D-panthenol (**B**) for 24 h, and the cell viability was determined using MTT assays. The microRNA (mRNA) expression of the natural moisturizing factors HAS-2, HAS-3, and AQP3 was measured in DTA- or D-panthenol-treated HaCaT cells using RT-PCR (**C**) and real-time PCR (**D**). D-panthenol was used as a positive control. (**E**) The protein expression of HAS-2 and HAS-3 in HaCaT cells treated with DTA for 24 h was measured using western blotting. * *p* < 0.05, ** *p* < 0.01 compared with control.

The cytotoxicity of DTA was investigated in human keratinocyte HaCaT cells using MTT assays. DTA was not cytotoxic at concentrations up to 25 μM (Figure 2A). To evaluate the effects of DTA on skin hydration, we first examined the microRNA (mRNA) expression of HAS-2, HAS-3, and AQP3, in DTA-treated HaCaT cells using RT-PCR. In here, we compared gene expression with D-panthenol, which is a provitamin B5 as a positive drug. D-panthenol is known to be effective for epidermal wound healing, anti-inflammation, and regenerating properties, and is widely used for various cosmetic products as a skin moisturizer [27,28]. The mRNA expression of HAS-2 and HAS-3 were upregulated by DTA in a dose-dependent manner, but AQP3 expression was not altered by DTA (Figure 2C). Particularly, DTA had a stronger regulatory effect on HAS-2 than D-panthenol. The upregulation of NMF synthase by DTA was confirmed using quantitative real-time PCR. DTA quantitatively increased the mRNA expressions of HAS-2, HAS-3, and AQP3 (Figure 2D). We further analyzed the skin-hydration effects of DTA at the protein level using western blotting. The protein expressions of HAS-2 and HAS-3 were significantly elevated by DTA (Figure 2E).

### 2.2. Effects of DTA in Keratinocyte Differentiation

**Figure 3:**
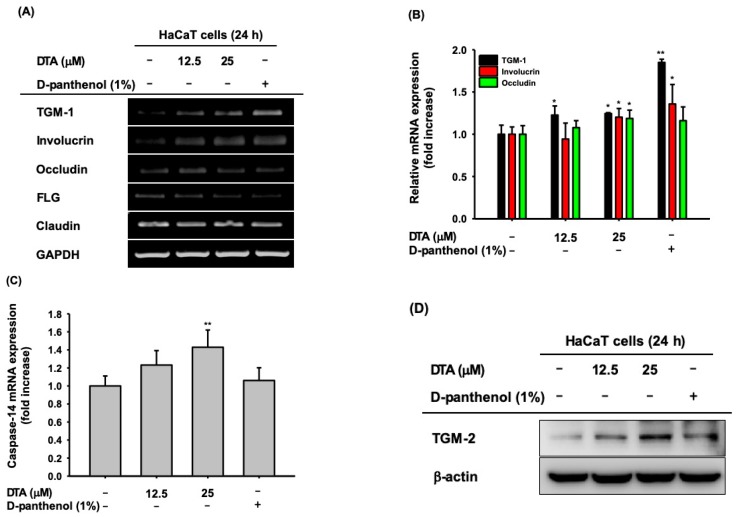
Effects of DTA on keratinocyte differentiation in HaCaT cells. (**A**) The mRNA expression of genes related to keratinocyte differentiation (TGM-1, involucrin, occludin, filaggrin (FLG), claudin) in HaCaT cells treated with DTA (0–25 μM) or D-panthenol (1%) was determined using RT-PCR. The mRNA expressions of TGM-1, involucrin, and occludin (**B**), as well as caspase-14 (**C**), were determined using real-time PCR. (**D**) The protein expression of TGM-2 was identified using Western blotting. * *p* < 0.05, ** *p* < 0.01 compared with control.

To analyze the effects of DTA on keratinocyte differentiation, the mRNA expression of various keratinocyte differentiation markers, including TGM-1, involucrin, and FLG, was measured in DTA-treated HaCaT cells using RT-PCR. DTA significantly increased TGM-1, involucrin, and occludin (Figure 3A); DTA did not regulate the mRNA expression of FLG or claudin. These regulatory effects of DTA were confirmed using quantitative real-time PCR (Figure 3B). In addition, DTA upregulated the mRNA expression of caspase-14 in a dose-dependent manner (Figure 3C). We further examined the effects of DTA on keratinocyte differentiation by western blotting. As expected, DTA strongly increased the protein expression of TGM-2 (Figure 3D).

### 2.3. Effects of DTA on the AP-1 Signaling Pathway

**Figure 4:**
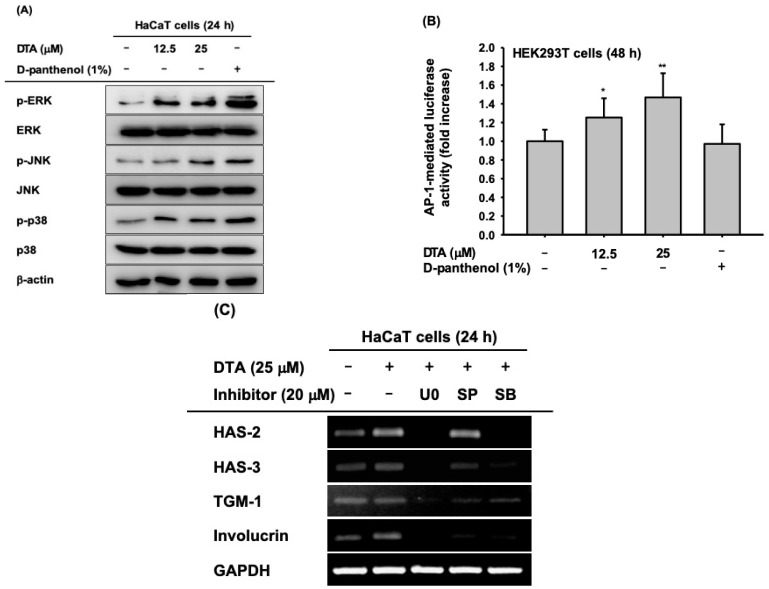
Effects of DTA on the AP-1 signaling pathway in HaCaT cells. (**A**) The levels of phosphorylated and total form of the mitogen activated protein kinases (MAPKs, ERK, JNK, and p38) in DTA- (0–25 μM) or D-panthenol-treated HaCaT cells were determined using immunoblotting. (**B**) HEK293T cells were transfected with plasmids expressing AP-1-luciferase (1 μg/mL) and -galactosidase in the presence of DTA (0–25 μM) or D-panthenol (1%) for 48 h, and AP-1 luciferase activity was determined by measuring luminescence. (**C**) The mRNA expression of HAS-2, HAS-3, TGM-1, and involucrin in HaCaT cells treated with DTA and MAPK inhibitors (U0126, SP600125, and SB203580) was determined using RT-PCR. * *p* < 0.05, ** *p* < 0.01 compared with control.

To investigate the regulatory mechanisms of DTA that promote skin hydration and differentiation in human keratinocytes, the activation of MAPKs, including ERK, JNK, and p38, was examined using western blotting. The phosphorylation of ERK, JNK, and p38 was significantly augmented by DTA in a dose-dependent manner (Figure 4A), and the enhancement of phosphorylation by DTA was similar to the effects of D-panthenol. Furthermore, we measured AP-1 promoter activity in DTA-treated HEK293T cells using a luciferase reporter assay. As expected, DTA dose-dependently increased AP-1 promoter activity (Figure 4B). To confirm that the effects of DTA occur through the AP-1 signaling pathway, the mRNA expression of hydration and differentiation markers were examined in HaCaT cells co-treated with DTA and MAPK inhibitors. The increased mRNA levels of HAS-2 and HAS-3 were suppressed by MAPK inhibitors (Figure 4C). In particular, U0126 strongly inhibited the gene expressions of HAS-2 and HAS-3. Moreover, MAPK inhibitors blocked the increased mRNA expression of TGM-1 and involucrin in co-treated HaCaT cells. 

### 2.4. Effects of DTA on the NF-κB Signaling Pathway

**Figure 5:**
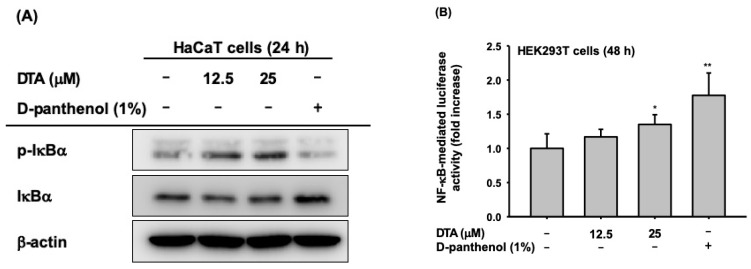

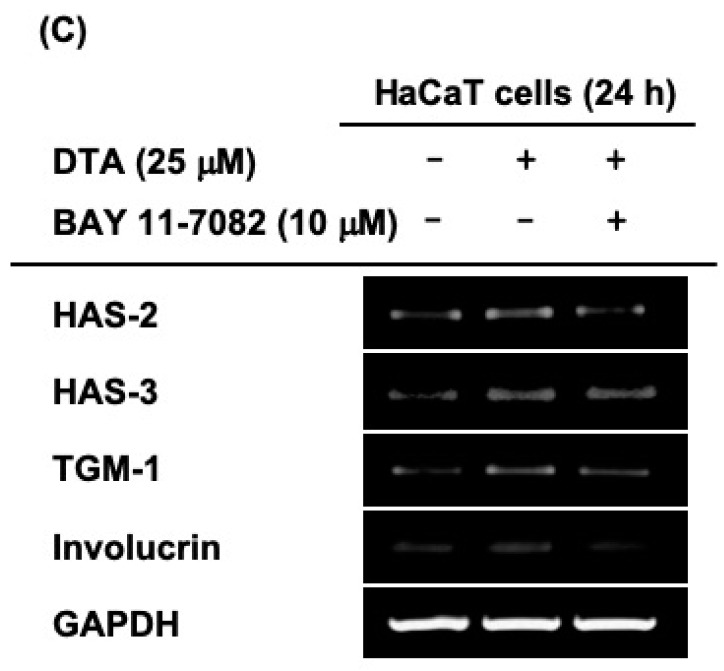
Effects of DTA on the NF-κB signaling pathway in HaCaT cells. (**A**) The levels of phosphorylated and total form of IκBα were determined using immunoblotting. (**B**) HEK293T cells were transfected with plasmids expressing NF-κB-luciferase (1 μg/mL) and β-galactosidase in the presence of DTA (0–25 μM) or D-panthenol (1%) for 48 h, and NF-κB luciferase activity was determined by measuring luminescence. (**C**) The mRNA expression of HAS-2, HAS-3, TGM-1, and involucrin in HaCaT cells treated with DTA or the IκBα inhibitor BAY 11-7082 was determined using RT-PCR. * *p* < 0.05, ** *p* < 0.01 compared with control.

Next, we examined whether the effects of DTA on skin hydration and differentiation were regulated through the NF-κB signaling pathway. The phosphorylation of IκBα, an inhibitor of NF-κB, was clearly increased by DTA (Figure 5A). Moreover, DTA clearly induced NF-κB promoter activity (Figure 5B). To confirm these results, the mRNA expression of genes involved in skin hydration and differentiation was investigated in HaCaT cells co-treated with DTA and IκBα inhibitors. The IκBα inhibitor BAY 11-7082 suppressed the expression of HAS-2, HAS-3, TGM-1, and involucrin that had been increased by DTA (Figure 5C).

## 3. Discussion

*P. cocos* Wolf (Polyporaceae) has been used as a traditional medicine for the treatment of diuretic, sedative, and tonic symptoms in China [25]. Dried sclerotia of *P. cocos* Wolf are used in the Chinese drug hoelen, which is used for treating diarrhea, kidney inflammation, and stomach problems. Moreover, these hyphae are used in combination with other drugs, such as DX-9386, Geiji-Bokryung-Hwan (GBH), and PAP 9704 [21,24,29]. DTA, one of the lanostane-type triterpene acids isolated from *P. cocos*, has antidiabetic and anti-cancer effects [25,30]; however, there are no reports on the effects of DTA on the skin. Therefore, this study aimed to demonstrate the effects of DTA on skin barrier function and its underlying molecular mechanisms.

Adequate moisturizing ability is vital to the skin’s barrier function [31,32]. The homeostasis of moisture must be maintained for the skin to function properly. In addition, water loss from the skin leads to factors of skin aging and damaged skin [33]. Another important reason to maintain skin hydration is the appearance of healthy and youthful skin. Therefore, moisturizing products are a major part of the cosmetics industry. Accordingly, with the recently increased interest in naturally derived materials, DTA might be a candidate for an effective and safe cosmetic ingredient because it is safely used in medicine and food.

HA produced by HAS is a representative molecule involved in skin moisturizing; therefore, increasing HA synthesis is an effective strategy for improving skin moisturization [34]. Another major factor in skin hydration is AQP3, an aquaporin transmembrane water channel that transports glycerol and water to regulate various physiological functions, such as cellular metabolism and skin hydration. In particular, AQP3 plays an important role in skin-moisture homeostasis by maintaining an osmotic gradient [14,35]. In this study, we examined the mRNA expression of HASs and AQP3 in DTA-treated HaCaT cells; as results, DTA upregulated gene expression of HAS-2, HAS-3, and AQP3. Interestingly, DTA increased the expression of these genes to a greater extent than D-panthenol, a well-known skin moisturizer [27,28]. We further confirmed that DTA upregulated the expression of HAS-2 and HAS-3 at the protein level using Western blotting. These results suggested that DTA can enhance the barrier function of the skin by upregulating moisturizing molecules.

Epidermal differentiation is an essential mechanism for skin hydration and barrier function [36,37]. Keratinocytes differentiate in the basal layer of the epidermis and migrate to the outer layer. The outermost layer of the epidermis, the stratum corneum, consists of corneocytes and prevents external irritation and water loss [38]. NMFs are abundant in corneocytes and contribute to skin moisturizing and barrier function. Since NMF-related genes include TGM, involucrin, and FLG, and these molecules are used as keratinocyte differentiation markers, the activation of these genes is required for skin function to be improved [17,18,39]. The mRNA level of TGM-1, involucrin, and occludin were increased by DTA. In addition, DTA enhanced the mRNA expression of caspase-14, which is involved in the terminal differentiation of keratinocytes. Moreover, we confirmed using immunoblotting that the expression of TGM-2 was dose-dependently increased by DTA. These results indicate that DTA strengthens the skin barrier by activating keratinocytes differentiation.

MAPKs, which are composed of the ERK, JNK, and p38 subfamilies, are involved in various cellular responses, such as cell proliferation and differentiation, through the AP-1 signaling pathway [19,40]. HAS is enhanced through MAPK and IκBα-signaling pathways [40,41], and TGM-1 is regulated by the p38/MAPK pathway and NF-κB signaling [42]. To determine the mechanism of DTA in skin hydration and keratinocyte differentiation, we measured the phosphorylation of MAPKs and AP-1-mediated luciferase activity in DTA-treated cells. DTA not only increased the phosphorylation of ERK, JNK, and p38, but also increased AP-1 promoter activity. Moreover, the increased mRNA expressions of HAS-2, HAS-3, TGM-1, and involucrin, following DTA treatment, were repressed by MAPK-specific inhibitors (U0126, SP600125, and SB203580). Similarly, DTA increased the phosphorylation of IκBα, as well as NF-κB luciferase activity. Thus, BAY 11-7082, an IκBα inhibitor, inhibited the increased mRNA expression of HAS-2 and TGM-1. Our results indicate that the skin moisturizing and keratinocyte-differentiation effects of DTA are mediated via upregulating the MAPK/AP-1 and IκBα/NF-κB pathways. However, further studies are required to clarify how DTA regulates the enzyme activity of MAPKs and IκBα. In addition, although we found that DTA improves in vitro skin barrier function in human keratinocytes cell line, further three-dimensional (3D) human skin model studies are separately required for DTA to be used as a cosmetic ingredient.

**Figure 6:**
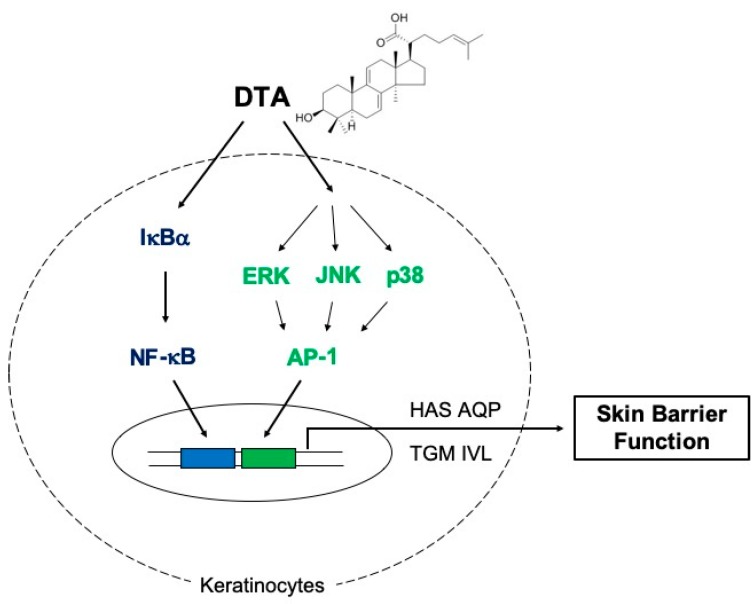
Schematic of DTA-mediated regulatory mechanisms for skin hydration and barrier function.

In summary, our results demonstrate that DTA enhances skin hydration and keratinocyte differentiation, in vitro, by activating the MAPK/AP-1 and IκBα/NF-κB signaling pathways (Figure 6). These findings suggest that DTA could be developed as an effective ingredient in cosmetics for moisturizing and skin barrier function. 

## 4. Materials and Methods

### 4.1. Materials

DTA was obtained from ChemFaces (Wuhan, China). D-panthenol was purchased from Sigma-Aldrich (St. Louis, MO, USA). Dulbecco’s modified Eagle’s medium (DMEM), fetal bovine serum (FBS), phosphate-buffered saline (PBS), penicillin, and streptomycin were purchased from HyClone (Grand Island, NY, USA). Sodium dodecyl sulfate (SDS), dimethyl sulfoxide (DMSO), and bovine serum albumin (BSA) were obtained from Sigma-Aldrich (St. Louis, MO, USA). Antibodies specific for each target protein were purchased from either Cell Signaling Technology (Beverly, MA, USA) or Santa Cruz Biotechnology (Santa Cruz, CA, USA). Primers specific for each target for semi-quantitative RT polymerase reaction (PCR) and quantitative real time PCR were synthesized at Bioneer Inc. (Daejeon, Korea).

### 4.2. Cell Culture

Human keratinocyte HaCaT cells purchased from the American Type Culture Collection (Rockville, MD, USA), were cultured in Dulbecco’s Modified Eagle Medium (DMEM), supplemented with 10% FBS and 1% penicillin-streptomycin at 37 °C in a humidified 5% CO_2_ incubator.

### 4.3. Cell Viability

The cell viability of HaCaT cells treated with DTA was determined using a conventional MTT assay as described previously [43]. Briefly, the HaCaT cells were seeded in 96-well plates overnight and incubated with DTA at 37 °C for 24 h. The MTT assay was then performed as above.

### 4.4. RT-PCR and Quantitative Real-Time PCR

To quantify the gene expression of skin barrier function-related factors, HaCaT cells were treated with either DTA or D-panthenol for 24 h. D-panthenol was used as a positive control. To determine the regulatory mechanisms of DTA on skin hydration and keratinocyte differentiation, HaCaT cells were pre-treated with DTA (25 μM) for 30 min and then treated with inhibitors including U0126 (20 μM), SP600125 (20 μM), SB203580 (20 μM), and BAY 11-7082 (10 μM). Then, total RNA was isolated using TRI reagent^®^ (Sigma-Aldrich, St. Louis, MO, USA), according to the manufacturer’s instructions. Complementary DNA was synthesized from total RNA using MuLV reverse transcriptase, according to the manufacturer’s instructions. Semi-quantitative RT-PCR and quantitative real-time PCR were conducted as previously described [15,44]. The primer sequences used in this study are listed in Table 1.

### 4.5. Western Blotting

HaCaT cells were seeded in six-well plates and treated with DTA (0–25 μM) or D-panthenol for 24 h. Total cell lysates were prepared by a lysis buffer (1 M Tris-HCl pH 7.5, 0.5 M NaF, 1 M -glycerol phosphate pH 7.5, 4 M NaCl, 100% NP-40, 2 μg/mL leupeptin, 2 μg/mL aprotinin, 2 μg/mL pepstatin A, 0.1 mM Na_3_VO_4_, 1 mM benzamide, 0.1 mM phenylmethanesulfonyl fluoride (PMSF), and 1.6 mM pervanadate), and subjected to SDS-polyacrylamide gel electrophoresis (SDS-PAGE) and transferred onto polyvinylidene fluoride membranes. Western blotting analysis was performed as previously reported [45]. The total or phosphorylated forms of signaling molecules (ERK, JNK, p38, and I B) and moisturizing-related proteins (HAS-2, HAS-3, and TGM-2) were detected using specific antibodies and visualized using chemiluminescence reagents. -Actin was used as an immunoblotting loading control.

### 4.6. Luciferase Reporter Gene Assay

To measure activities of the AP-1- and NF- B-promoter, HEK293T cells were seeded at 1.2 × 10^5^ cells/well in 24-well plates. After 24 h, cells were transfected with 0.8 μg/mL of plasmids containing -galactosidase and NF- B-Luc or AP-1-Luc for 24 h. Transfection was conducted using the polyethylenimine (PEI) method. The cells were treated with DTA (0–25 μM) for an additional 24 h. Luciferase assay was conducted using the Luciferase Assay System (Promega, Madison, WI, USA). Luciferase activity was normalized to -galactosidase activity.

### 4.7. Statistical Analysis

All results are expressed as the mean ± standard deviation (SD) of at least three independent experiments. For statistical analysis, the data were compared between experimental groups using the Mann–Whitney *U* test or analysis of variance (ANOVA). The *p*-Values < 0.05 were considered statistically significant, and the statistical comparisons were analyzed using SPSS software (SPSS Inc., Chicago, IL, USA).

## Figures and Tables

**Table 1:** Primer sequences used in this study.

Name		Sequences (5′ to 3′)
Semi-quantitative RT-PCR		
HAS-2	F	CTGCTTGACCCTCAGAGACC
	R	CTGCTTGACCCTCAGAGACC
HAS-3	F	GCCACTTGTCGGCGATAAGG
	R	CACTGTCCACCCCTCAGAGC
AQP3	F	CAAAAGCTGGGAAGCCTTCT
	R	CCATCCTTCAAAAGGCGCAG
TGM-1	F	CCGACCACCACTACAGCAAG
	R	GGGCAGGGAACCAGCATCTT
Involucrin	F	AAGGTGCAGTTTTGCCAAGG
	R	CAACCCTCTGCACCCAGTTT
Occludin	F	CACTCACGGCAAATTCAACGGCAC
	R	GACTCCACGACATACTCAGCAC
FLG	F	CACTCACGGCAAATTCAACGGCAC
	R	GACTCCACGACATACTCAGCAC
Claudin	F	CACTCACGGCAAATTCAACGGCAC
	R	GACTCCACGACATACTCAGCAC
GAPDH	F	CACTCACGGCAAATTCAACGGCAC
	R	GACTCCACGACATACTCAGCAC
Quantitative real-time PCR		
HAS-2	F	GTCCCTACCGAGTCTCTTCT
	R	TTTTTAAGTTTCCGCTTCTG
HAS-3	F	GGTTGGACCTACAAGGAGGC
	R	GGTTCATGCTGGTGTCCTCA
AQP3	F	TACCCCCAGGAGAAGATTCC
	R	TTTTCTGCCAGTGCCTCTTT
TGM1	F	GAAAGCATGATCCGGGACGT
	R	GATGGCAGAGAGGAGGTTGA
Involucrin	F	CCAACGCAAAGCAATACATGA
	R	CCTTTTTCGCTTCCCTGTTTTA
Occludin	F	CCAACGCAAAGCAATACATGA
	R	CCTTTTTCGCTTCCCTGTTTTA
Caspase-14	F	CCAACGCAAAGCAATACATGA
	R	CCTTTTTCGCTTCCCTGTTTTA
GAPDH	F	CAATGAATACGGCTACAGCAAC
	R	AGGGAGATGCTCAGTGTTGG
